# Long-term Emergency Department Visits and Readmissions After Laparoscopic Roux-en-Y Gastric Bypass: a Systematic Review

**DOI:** 10.1007/s11695-021-05286-0

**Published:** 2021-04-04

**Authors:** N. van Olst, A. S. van Rijswijk, S. Mikdad, L. J. Schoonmade, A. W. van de Laar, Y. I. Z. Acherman, S. C. Bruin, D. L. van der Peet, L. M. de Brauw

**Affiliations:** 1 Department of Surgery, Spaarne Gasthuis, Spaarnepoort 1, 2134 TM Hoofddorp, The Netherlands; 2 Department of Surgery, Amsterdam UMC, De Boelelaan 1117, 1081 HV Amsterdam, The Netherlands; 3 Medical Library, Vrije Universiteit Amsterdam, De Boelelaan 1117, P.O. Box 7057, 1007 MB Amsterdam, The Netherlands

**Keywords:** Roux-en-Y gastric bypass, Emergency department visits, Readmissions

## Abstract

**Purpose:**

There is considerable evidence on short-term outcomes after laparoscopic Roux-en-Y gastric bypass (LRYGB), but data on long-term outcome is scarce, especially on postoperative emergency department (ED) visits and readmissions. We aim to systematically review evidence on the incidence, indications, and risk factors of ED visits and readmissions beyond 30 days after LRYGB.

**Materials and Methods:**

A systematic search in PubMed, Scopus, Embase.com, Cochrane Library, and PsycINFO was performed. All studies reporting ED visits and readmissions > 30 days after LRYGB, with ≥ 50 patients, were included. PRISMA statement was used and the Newcastle-Ottawa Scale for quality assessment.

**Results:**

Twenty articles were included. Six studies reported on ED visits (*n* = 2818) and 19 on readmissions (*n* = 276,543). The rate of patients with an ED visit within 90 days after surgery ranged from 3.9 to 32.6%. ED visits at 1, 2, and 3 years occurred in 25.6%, 30.0%, and 31.1% of patients. Readmissions within 90 days and at 1-year follow-up ranged from 4.1 to 20.5% and 4.75 to 16.6%, respectively. Readmission was 29% at 2 years and 23.9% at 4.2 years of follow-up. The most common reason for ED visits and readmissions was abdominal pain.

**Conclusion:**

Emergency department visits and readmissions have been reported in up to almost one in three patients on the long-term after LRYGB. Both are mainly indicated for abdominal pain. The report on indications and risk factors is very concise. A better understanding of ED visits and readmissions after LRYGB is warranted to improve long-term care, in particular for patients with abdominal pains.

**Supplementary Information:**

The online version contains supplementary material available at 10.1007/s11695-021-05286-0.

## Introduction

Bariatric surgery has gained ground due to the increasing prevalence of obesity over the past decades [1–3]. Worldwide, the number of bariatric surgeries performed nearly doubled between 2008 and 2016 and approached 700,000 procedures in 2016 [4]. The laparoscopic Roux-en-Y gastric bypass (LRYGB) is still one of the most commonly performed bariatric surgeries in the world [4, 5]. It induces sustainable weight loss, and has proven to be a viable solution for obesity-related comorbidities and reduces overall mortality [6–10]. The LRYGB can be considered safe, with acceptable morbidity and mortality rates within 30 days of surgery [6, 9].

Two quality measures after LRYGB surgery are emergency department (ED) visits and readmissions. These are considered to be markers of poor coordination of care and provide insight in the complications during post-bariatric care. They also reflect the long-term impact that a given intervention may have on public health, and healthcare systems, with direct and indirect financial consequences. There is extensive evidence on the short-term outcomes after LRYGB with 5.1–6.1% readmissions and 11.3% ED visits in the first 30 days after surgery [11–13]. The most frequently met long-term complications are internal herniation, ulcers at the gastrojejunal anastomosis, and cholelithiasis [14–18]. Also complaints of diarrhea, fatigue, anemia, hypoglycemia, and dumping are often present [19]. The magnitude and impact of these long-term complaints are less evident.

With a growing number of bariatric surgeries, improvement of long-term care after LRYGB is necessary. The primary aim of this study is to review the current literature focusing on the number and reasons for ED visits and readmission > 30 days after LRYGB. Insight in occurrence, diagnostics, and treatment of long-term complaints is essential. Therefore, it is important to score reasons for long-term ED visits and readmissions.

## Materials and Methods

### Data Sources

This systematic review was conducted in accordance with the Preferred Reporting Items for Systematic Reviews and Meta-Analysis (PRISMA) statement [20]. A comprehensive search was performed, in collaboration with a medical librarian (LS), in the bibliographic databases PubMed, Embase.com, PsycINFO (via Ebsco), the Cochrane Library, and Scopus from inception to October 17, 2019. Search terms included controlled terms (MeSH in PubMed, Emtree in Embase, and thesaurus terms in PsycINFO) as well as free-text terms. The following terms were used, including synonyms and closely related words, as index terms or free-text words: “bariatric surgery” and “readmission” or “emergency department visit.” The search was performed without date or language restrictions. Furthermore, the references of the included articles were manually screened for cross-references. The full search strategies for all databases can be found in Supplement 1.

### Main Outcomes and Measures

The primary outcome measure of this study is the number of ED visits and readmissions > 30 days after LRYGB. The secondary outcome measures are indications and risk factors for ED visits and readmissions.

### Study Selection

Duplicate articles have been manually removed. Unique articles were screened by title and abstract by two independent reviewers (N.O. and S.M.), who also performed the full text screening, data extraction, and methodological quality assessment. Discrepancies were resolved by consensus or a third party (M.B.) if necessary.

Full texts of the selected articles were screened for eligibility. Inclusion criteria were as follows: (1) randomized controlled trials, prospective and retrospective cohort, and case-control studies; (2) articles with at least 50 patients included with a RYGB of which at least 80% laparoscopic and a maximum of 20% open surgeries; (3) data on ED visits and readmissions > 30 days after LRYGB.

Exclusion criteria were as follows: (1) case reports, review articles, commentaries, letters to editors, abstracts only; (2) studies in other languages than English; (3) studies that report on revisional or robotic RYGB; (4) articles which only included children or adolescents.

The following data was extracted from the included articles using a predesigned extraction form: first author’s family name, country, study design, time of operation, number of patients, demographic and clinical characteristics of patients (gender, age, preoperative BMI), duration of follow-up (FU), number of ED visits and/or readmissions, and indications and risk factors for ED visits and readmissions.

### Quality Assessment

The methodological quality of the studies was assessed using the Newcastle-Ottawa scale (NOS) [21]. The NOS applies to observational studies only (cohort and case-control studies). This scale contains eight items belonging to three categories: (1) study group selection, (2) comparability of groups, and (3) outcome/exposure of interest. A study with a total score > 7 was judged to be of high quality [21].

### Statistics

Descriptive statistics are used. Data is presented in numbers and percentages, as mean or median with standard deviation (SD) or interquartile ranges, unless otherwise described. A meta-analysis could not be performed due to heterogeneity in the study characteristics and outcomes.

## Results

### Study Selection

**Fig. 1:**
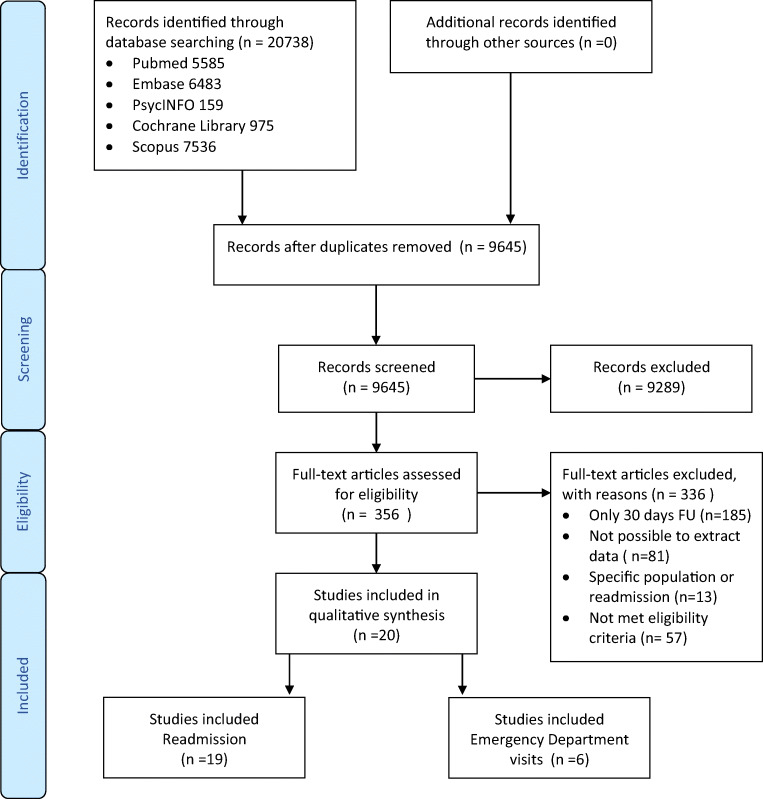
Flow chart of the study selection

The search strategy resulted in 9645 unique citations after excluding duplicates. Title and abstract were screened, and 356 articles were extracted of which the full texts were reviewed for eligibility. A total of twenty studies were included. Of these studies, six reported on ED visits [22–27] and nineteen on readmissions [23, 24, 26–42]. Figure 1 provides an overview of our literature search and study selection.

### Study and Patient Characteristics

Study and patient characteristics and data on FU of all included studies are presented in Table 1. All included studies were retrospective cohort studies and case-control studies, published between 2008 and 2019. The FU for ED visits ranged between 60 days and 3 years and for readmissions between 6 weeks and 4.2 years after surgery.

**Table 1:** Study and patient characteristics of included studies

Study characteristics				Duration of follow-up				Outcome measures		Patient characteristics		
Study	Country	Year of operation	LRYGB included, *n*	90 days	1 year	2 years	Others	Readmission	ED visits	Female % (*n*)	Preoperative BMI in kg/m^2^	Age in years
Arterburn, 2014 [37]	USA	1/1/2005–31/12/2009	5950				Median 1.5 years	√		83.5% (4968)	44.6 (44.4–44.7) Mean (95%CI)	45.8 (45.6–46.1) Mean (95%CI)
Celio, 2016 [38]	USA	2007–2012	42119	√				√		74.3% (31294)	56.4 (55.7–56.4) Mean (95%CI)	43.8 (43.6–43.9) Mean (95%CI)
Celio, 2017 [39]	USA	2007–2012	135040	√				√		78.5% (106006)	47.5 (8.3) Mean (SD)	45.4 (11.7) Mean (SD)
Cho, 2008 [25]	USA	1/2001 to 3/2004	733				3 years		√	82.9% (608)	49.7 (30–97) Mean (range)	44.7 (21–76) Mean (range)
Garg, 2016 [40]	USA	2003–2013	1352		√			√		ND	ND	ND
Gero, 2019 [41]	Switzerland	1/6/2012–31/5/2017	4120		√			√		80.1% (3302)	41.3 (6.2) Mean (SD)	38.2 (11.1) Mean (SD)
Gribsholt, 2016 [32]	Denmark	1/1/2006–31/12/2010	9895				Mean 4.2 years	√		78% (7718)	ND	40.2 (33.5–47.1) median (range)
Inabnet, 2011 [33]	USA	6/2007–11/2010	14329	√				√		ND	ND	ND
Kellogg, 2009 [26]	USA	1/8/2004–31/5/2007	1222	√				√	√	80% (977)	50.0 (35–96) Mean (range)	42 (15–71) Mean (range)
Kizy, 2017 [34]	USA	2012–2015	33657	√				√		79.0% (26602)	ND	44.9 (12) Mean (SD)
Li, 2015 [42]	USA	1/1/2010–31/12/2011	1313		√			√		82.9% (1089)	42.2 (6.4) Mean (SD)	43.9 (10.4) Mean (SD)
Shah, 2016 [28]	USA	1/1/2010–7/3/2014	270	√				√	√	81.5% (220)	47.0 Mean	47.3 Mean
Telem, 2014 [29]	USA	2006–2008	12439			√		√		ND	ND	ND
Waydia, 2014 [35]	United Kingdom	9/2010–10/2013	123				6 weeks	√		87.3% (107)	46.6 (5.9) Mean (SD)	46.7 (27–69.5) Mean (range)
Groups												
Chen 2016 [23]	USA	1/1/2010–1/5/2013	Total 132	√				√	√	81.8% (108)		
			Medicaid 33							90.9% (30)	49.6 Mean	39.0 Mean
			Non-Medicaid 99							78.8% (78)	47.1 Mean	48.7 Mean
Dallal, 2008 [27]	USA	9/2003–11/2006	Total 341				60 days	√	√	78% (266)		
			Private practice 217							77% (167)	46 (32–82) Mean (range)	43.7 (15–74) Mean (range)
			Academic 124							80% (99)	47 (35–75) Mean (range)	42.4 (16–70) Mean (range)
Funk, 2014 [24]	USA	1/2008–4/2011	Total 120	√				√	√	75.0% (90)		
			Medicaid 30							63.3% (19)	58.4 Mean	42.0 Mean
			Non-Medicaid 90							78.9% (71)	49.5 Mean	47.6 Mean
Major, 2017 [30]	Poland	4/2013–6/2016	Total 198	√				√		63.0% (124)		
			Circular stapled 99							63.0% (62)	42.5 (40.4–45.6) Median (IQR)	48 (41–53) Median (IQR)
			Linear stapled 99							63.0% (62)	42.7 (40.5–45.7) Median (IQR)	47 (40–53) Median (IQR)
Rogula, 2018 [31]	USA	1/2008–12/2015	Total 253		√			√				
			Hand sewn 21							ND	45.4 (5.6) Mean (SD)	46.5 (13.6) Mean (SD)
			Circular stapled 82							ND	48.0 (7.0) Mean (SD)	42.0 (11.8) Mean (SD)
			Linear stapled 150							ND	48.5 (7.0) Mean (SD)	44.3 (12.0) Mean (SD)
Roy, 2017 [36]	USA	1/1/2012–30/9/2015	Total 13670	√				√		78.6% (10751)		
			Powered stapler 4057							81.0% (3286)	ND	44.9 (11.7) Mean (SD)
			Manual stapler 9613							77.7% (7465)	ND	45.8 (11.8) Mean (SD)

*BMI* body mass index, *ED visits* emergency department visits, *LRYGB* laparoscopic Roux-en-Y gastric bypass, *ND* no data, *IQR* interquartile range, *SD* standard deviation, *95%CI* 95% confidence interval

Most studies were single-arm studies that reported outcomes only after LRYGB or two-armed studies involving different types of bariatric surgeries. Others were two-armed studies comparing results between Medicaid and non-Medicaid patients [23, 24], patients in which a different technique was used to close the gastrojejunostomy [30, 31, 36], or compared private practice versus an academic hospital [27].

The included studies comprised a total of 2818 patients with data about ED visits and 276,543 with data about readmissions. In all studies, the majority of patients were female (63.0–90.9%), mean body mass index (BMI) ranged from 41.3–58.4 kg/m^2^ and mean age from 38.2 to 48.7 years.

### Methodological Quality Assessment

According to the NOS, only nine studies were of high quality [27, 28, 30–33, 36, 37, 39]. Most studies missed points due to the fact that they did not match patients or did not make any adjustment for confounders in the analysis. The outcome of the quality assessment is shown in Table 2.

**Table 2:** Methodological quality assessment using the Newcastle-Ottawa scale

**Study, year**	**Selection**	**Comparability**	**Outcome**	**Total**
Arterburn, 2014 [37]	***	**	***	********
Celio, 2016 [38]	***	**	**	******
Celio, 2017 [39]	***	.	***	*******
Cho, 2008 [25]	***	.	**	*****
Dallal, 2008 [27]	**	**	***	*******
Garg, 2016 [40]	***	.	**	*****
Gero, 2019 [41]	***	.	***	******
Gribsholt, 2016 [32]	***	**	***	********
Inabnet, 2011 [33]	***	**	**	*******
Kellogg, 2009 [26]	***	.	**	*****
Kizy, 2017 [34]	***	.	**	*****
Li, 2015 [42]	***	.	***	******
Rogula, 2018 [31]	***	**	**	*******
Roy, 2017 [36]	***	**	**	*******
Shah, 2016 [28]	***	**	***	********
Telem, 2014 [29]	***	.	**	*****
Waydia, 2014 [35]	***	.	**	*****
**Study, year**	**Selection**	**Comparability**	**Exposure**	**Total**
Chen 2016 [23]	**	.	***	*****
Funk, 2014 [24]	**	.	***	*****
Major, 2017 [30]	***	**	**	*******

A study with a total score > 7* was judged to be of high quality

### Emergency Department Visits

An overview of the results on ED visits is presented in Table 3. Sixty days after surgery, the range for ED visits was 3.4–7.6% [26, 27]. The percentages of patients with an ED visit within 90 days after surgery ranged from 3.9 to 32.6% [23, 24, 26, 28]. Only one study by Cho et al. reported ED visits beyond 90 days. At 1 year, 2 years, and 3 years [25] of FU, rates of ED visits were 25.6%, 30%, and 31.1% respectively.

**Table 3:** Number of emergency department visits

Study, year	Patients included, *n*	ED visits 60 days % (*n*)	ED visits 90 days % (*n*)	ED visits 1 years % (*n*)	ED visits 2 years % (*n*)	ED visits 3 years % (*n*)
Cho, 2008 [25]	733			25.6% (188)	30% (220)	31.1% (228)
Kellogg, 2009 [26]	1222	3.4% (41)	3.9% (48)			
Shah, 2016 [28]	270		27.1% (73)			
Groups						
Chen 2016 [23]	Total 132		32.6% (43)			
	Medicaid 33		48.2% (16)			
	Non-Medicaid 99		27.4% (27)			
Dallal, 2008 [27]	Total 341	7.6% (26)				
	Private practice 217	1.4% (3)				
	Academic 124	18.5% (23)				
Funk, 2014 [24]	Total 120		15.8% (19)			
	Medicaid 30		33.3% (10)			
	Non-Medicaid 90		10.0% (9)			

*ED visits* emergency department visits

The indications for ED visits are presented in Table 4. The main indications for ED visits at 90 days of FU were nausea and vomiting, dehydration, abdominal pain (cholelithiasis not included), and wound issues [26]. At 1, 2, and 3 years of FU, main reasons were abdominal pain (45.2%, 47.4%, 47.6%), nausea and vomiting (18.4%, 17.9%, 18.4%), abdominal pain and vomiting combined (16.7%, 15.6%, 15.1%), and other complaints (19.7%, 19.2%, 19.0%) [25].

**Table 4:** Indications for emergency department visits

Study, year	Indications for ED visits in 90-day FU	Indications for ED visits in 1-year FU	Indications for ED visits in 2-year FU	Indications for ED visits in 3-year FU
Cho, 2008 [25]		Abdominal pain 45.2%	Abdominal pain 47.4%	Abdominal pain 47.6%
		Other complaints 19.7%	Other complaints 19.2%	Other complaints 19.0%
		Nausea and vomiting 18.4%	Nausea and vomiting 17.9%	Nausea and vomiting 18.4%
		Abdominal pain and vomiting 16.7%	Abdominal pain and vomiting 15.6%	Abdominal pain and vomiting 15.1%
Kellogg, 2009 [26]	Nausea/vomiting			
	Dehydration			
	Abdominal pain (without cholelithiasis)			
	Wound issues			

*ED visits* emergency visits, *FU* follow-up

In addition, Cho et al. showed that 32.5% of all patients with an ED visit had more than 2 visits [25].

Funk et al. and Dallal et al. showed that Medicaid patients had significantly more ED visits than non-Medicaid patients [24, 27]. Although not statistically significant (*p* = 0.06), the ED visits reported in the study by Chen et al. were nearly twice as common in Medicaid patients compared to non-Medicaid patients (48.2% vs 27.4%) [23]. Patients who underwent open surgery or patients who were unemployed, disabled, or retired were at higher risk for ED visits [26]. Furthermore, undergoing surgery at an academic hospital was associated with a higher risk of visiting the ED after LRYGB [27].

### Readmissions

An overview of the results on readmissions is presented in Table 5. Within the first 90 days after surgery, the rates of readmission were between 4.1 and 20.5% [23, 24, 26, 28, 30, 33, 34, 36, 38–41]. The number of patients readmitted within a 1-year FU ranged from 4.75 to 16.6% [31, 40–42]. One and a half year readmission was 19.9% [37] and 2-year readmission was 29% [29]. The study of Gribsholt et al. showed a readmission of 23.9% with a mean FU of 4.2 years [32].

**Table 5:** Number of readmissions

Study, year	Patients included, *n*	Readmission 60 days % (*n*)	Readmission 90 days % (*n*)	Readmission 1 year % (*n*)	Readmission 2 years % (*n*)	Time of FU: readmission % (*n*)
Arterburn, 2014 [37]	5800					1.5 years: 19.9% (1155)
Celio, 2016 [38]	38035		9.2% (3499)			
Celio, 2017 [39]	135040		6.6% (8913)			
Garg, 2016 [40]	1352		6.14% (83)	7.17% (97)		180 days: 6.2% (84)
Gero, 2019 [41]	4120 ( 90 days) 3399 (1 year)		4.1%(169)	9.4% (320)		
Gribsholt, 2016 [32]	9895					Mean 4.2 IQR 3.5–5.3: 23.9% (2367)
Inabnet, 2011 [33]	14329		7.6% ( 1094)			
Kellogg, 2009 [26]	1222	8.3% (102)	10.3% (126)			
Kizy, 2017 [34]	33657		7.8% (2625)			
Li, 2015 [42]	1052			4.75% (50)		
Shah, 2016 [28]	270		12.3% (33)			
Telem, 2014 [29]	12439				29% (3607)	
Waydia, 2014 [35]	123					6 weeks: 6.5% (8)
Groups						
Chen 2016 [23]	Total 132		20.5% ( 27)			
	Medicaid 33		37% (12)			
	Non-Medicaid 99		14.7% (15)			
Dallal, 2008 [27]	Total 341	4.7% (16)				
	Private practice 217	1.4% (3)				
	Academic 124	10.4% (13)				
Funk, 2014 [24]	Total 120		10% (12)			
	Medicaid 30		20% (6)			
	Non-Medicaid 90		6.7% (6)			
Major, 2017 [30]	Total 198		7.1% (14)			
	Circular stapled 99		6.1% (6)			
	Linear stapled 99		8.2% (8)			
Rogula, 2018 [31]	Total 253			16.6% (42)		
	Hand sewn 21			14% (3)		
	Circular stapled 82			28% (23)		
	Linear stapled 150			11% (16)		
Roy, 2017 [36]	Total 13670	Total 7.2% ( 978)	Total 7.9% (1076)			
	Powered stapler 4057	6.8% (274)	7.6% (308)			
	Manual stapler 9613	7.3% (704)	8.0% (768)			

*IQR* interquartile range

The indications for readmissions and number of readmissions are presented in Table 6. Four studies showed indications for readmissions [26, 32, 40, 41]. The different timepoints of FU have shown similar reasons for readmissions in which abdominal pains, intestinal obstruction, gastrointestinal complaints including nausea and vomiting, and dietary complaints were most common.

**Table 6:** Additional information about readmissions

**Indications for readmission**			
**Study, year**	**Indications for readmission in 90-day FU**	**Indications for readmission in 1-year FU % (*n*)**	**Indications for readmission in 4.2 years (3.5–5.3) mean (IQR) FU**
Garg, 2016 [40]		GI:** 36.1% (35)	
		Dietary:* 29.9% (29)	
		VTE: 9.28% (9)	
		Bleed: 8.25% (8)	
		Pulmonary: 5.15% (5)	
		SSI/wound/abscess: 5.15% (5)	
		Anastomotic leak: 4.12% (4)	
		Other: 2.06% (2)	
Gero, 2019 [41]	Abdominal pain of unknown origin	Abdominal pain of unknown origin	
	Dysphagia	Symptomatic cholecytolithiasis	
	Internal herniation/bowel obstruction	Internal herniation/bowel obstruction	
Gribsholt, 2016 [32]			Abdominal pain 62.8%
			Intestinal obstruction 21.7%
Kellogg, 2009 [26]	Nausea/vomiting		
	Dehydration		
	Abdominal pain (without cholelithiasis)		
	Wound issues		
**Number of readmissions**			
**Study, year**	**Number of readmissions**	**Readmission 2 year % (*n*)**	
Telem, 2014 [29]	1	19% (2363)	
	2 or 3	8% (995)	
	4 or more	2% (249)	

*Vitamin deficiency, dehydration, elektrolyte imbalance

**Ulcers, strictures, and bowel obstruction

*GI* gastro-intestinal, *VTE* venous thromboembolism, *SSI* surgical site infection

The study by Telem et al. provides an overview on the number of readmissions per patient within a 2-year FU. Of all included patients, there was one readmission in 19% (2363), two to three readmissions in 8% (995), and four or more readmissions in 2% (249) [29].

The same risk factors as for ED visits apply to readmissions: open surgery, unemployment, being disabled, retirement [26], Medicaid status, or undergoing surgery at an academic hospital [27].

## Discussion

This systematic review is the first overview of long-term ED visits and readmissions after LRYGB. Available literature indicates that ED visits and readmissions after LRYGB are frequently seen. With regard to ED visits, one in three patients had at least one visit in the first 90 days after surgery [23, 24, 26, 28]. Remarkably, in our extensive search, we only found one study describing ED visits beyond 90 days. ED visits showed an increase over time with up to 31.1% of patients visiting the ED within 3 years after surgery [25]. The FU for the included studies with data on readmissions was up to 4.2 years post-surgery. Up to one in five patients was readmitted at least once within the first 90 days after surgery [23, 24, 26, 28, 30, 33, 34, 36, 38–41]. One-year readmission rate was up to 16.6%, 2-year readmission rate was 29%, and the percentage after 4.2 years of FU was 23.9% [29, 31, 32]. Five studies provided an overview of indications for ED visits and readmissions of which abdominal pain, intestinal obstruction, gastrointestinal complaints including nausea and vomiting, and dietary complaints were most common [25, 26, 32, 40, 41]. Open surgery, unemployment, being disabled, retirement, Medicaid status, or undergoing surgery at an academic hospital were risk factors for ED visits and readmissions [23, 24, 26, 27]. But only little or no data on risk factors was found for both of our outcome measures. Only nine studies in the current literature had a FU beyond 90 days. In the context of the increase in bariatric surgery over the past three decades, it is striking to find this little evidence on long-term ED visits and readmissions.

Despite the sparse number of articles providing reasons for ED visits and readmissions, abdominal pain is a frequently reported indication [25, 26, 32, 41]. Abdominal pain can be caused by some well-known reasons: internal herniation, ulcers at the gastrojejunal anastomosis, and cholelithiasis [14–18]. However, the burden of nonspecific complaints is considerable [25, 41]. The impact and extent of abdominal complaints is gaining more attention in the current literature [43]. In the article by Gormsen et al., 39% of patients experienced abdominal pain in the past month, within a FU of 2–7 years after RYGB [44]. In a survey by Gribsholt et al., 54.4% of patients experienced abdominal pain, 34.2% had abdominal pain leading to health care contact, and 18.5% had abdominal pain leading to hospitalization (symptoms of cholelithiasis and urolithiasis excluded) [19]. In the studies by Gormsen and Gribsholt, risk factors for abdominal pain were unemployment, retirement, women, patients younger than 35, symptoms before LRYGB, postoperative complications, preoperative smoking, and preoperative use of strong analgesics [19, 44]. Unemployment and retirement are consistent with the risk factors for ED visits and readmissions reported by Kellog et al. [26]. Hogestol et al. reported chronic abdominal pain in 33.8% of patients, indigestion in 48.8%, and irritable bowel syndrome in 29.1%. Mean FU was 64 months (SD 4.2) [45]. These data could confirm the fact that gastrointestinal complaints are another frequently mentioned cause for readmissions [40]. Future studies should elaborate on abdominal complaints in particular. Insight in ED visits and readmissions could provide more information on the impact, and the course of abdominal complaints through time after LRYGB.

It is common for one patient to have multiple ED visits or readmissions after LRYGB. The article by Cho et al. showed that, of all patients with an ED visit, 32.5% had more than 2 visits [25]. In the study by Telem et al., 10% of all patients had 2 or more readmissions within 2 years of FU [29]. Furthermore, a wide variation was seen in the number of readmissions between patients. One patient had 22 readmissions [29]. Multiple visits could be the cause of a previously unresolved or chronic complaint. A lot of complaints could be present after LRYGB like diarrhea, fatigue, anemia, hypoglycemia, dumping, and cholelithiasis [19]. However, some complaints are more likely to occur on the short term after LRYGB due to adjustments a patient has to make after surgery, like dumping syndrome, and dehydration. Later on, diagnoses such as anemia and cholelithiasis are expected to appear more frequently.

The wide variation of the results in this review is remarkable, for example, the range from 4.1 to 20.5% for readmissions within 90 days of FU [23, 41]. These differences can be attributed to heterogeneity in the definitions used for ED visits and readmission in these studies. Some studies show all causes for ED visits and readmissions [29, 37, 46, 47] whereas Kellog et al. and Gribsholt et al. only report surgery-related data [26, 32]. Other studies do not specify whether they are related to surgery or not. This could explain, for instance, why Telem et al. reported a higher rate of readmissions at 2 years FU, than Gribsholt et al. at 4.2 years of FU [29, 32]. This makes it difficult to draw conclusions from the results.

Due to the heterogeneity in reported data, pooling of data and meta-analysis was not possible. Underlying causes of these variations are the retrospective design of all studies; wide variation in confounding factors in case of baseline criteria like gender, age, and BMI; variation in the definitions used for ED visits and readmission; and great diversity in times of FU. An important limitation of our study is that we could only include retrospective studies. The evidence is subject to the disadvantages of retrospective data and selection bias. Another limitation is the coding for the types and approaches of bariatric surgeries over time. Since separate codes for laparoscopic procedures did not yet exist, it is not inconceivable that coding in the early stages of procedures included the open approach. This could have caused publication of biased results in these studies. The third limitation was already mentioned in the “Methodological Quality Assessment” section, as most of the studies lack information about confounding. Finally, the biggest confounder in this systematic review is the variation in the definitions used for ED visits and readmission.

## Conclusion

The number of ED visits and readmissions shows the severity of the long-term problems faced by bariatric patients and caregivers after LRYGB. While abundant evidence shows limited ED visits and readmissions in the first 30 days, this review shows ED visits and readmissions in nearly one in three patients long term after LRYGB. We can conclude that the majority of all ED visits and readmissions will take place longer than 30 days after surgery. This demonstrates that ED visits and readmissions, in the first 30 days after surgery, do not adequately reproduce the magnitude of postoperative problems faced by patients after LRYGB. Therefore, long-term FU after LRYGB should extend well beyond the first 30 postoperative days

More long-term prospective data after LRYGB is needed. Understanding the long-term problems after bariatric surgery is crucial for optimizing the choice for a specific procedure and long-term post-bariatric care.

Abdominal pain is the main reason for ED visits and readmissions after LRYGB. Insight into ED visits and readmissions could provide more information about the impact and course of abdominal complaints over time after LRYGB.

## Supplementary Information

ESM 1 (DOCX 15 kb)
