# Supplementary material for: Long-term Emergency Department Visits and Readmissions After Laparoscopic Roux-en-Y Gastric Bypass: a Systematic Review
Source: Obes Surg. 2021 Apr 4;31(6):2380–90. doi: 10.1007/s11695-021-05286-0 (PMC8113200; doi:10.1007/s11695-021-05286-0)
Supplement: Supplementary file 1 — (DOCX 15 kb) [file 11695_2021_5286_MOESM1_ESM.docx]

**Supplement 1: search strategies for all databases**

# **Results 17 oktober 2019**

| **Databases** | **Items found** |
| --- | --- |
| Pubmed | 5585 |
| Embase.com | 6483 |
| PsycINFO (Ebsco) | 159 |
| Cochrane Library (Wiley) | 975 |
| Scopus | 7536 |
| Total | 20738 |

## **History Pubmed October 17, 2019**

| Search | Pubmed Query - October 17, 2019 | Items found |
| --- | --- | --- |
| #3 | #1 AND 2 | 5585 |
| #2 | "Hospitalization"[Mesh] OR "Emergency Service, Hospital"[Mesh] OR "Patient Readmission"[Mesh] OR hospital*[tiab] OR rehosp*[tiab] OR readmission*[tiab] OR emergency service*[tiab] OR emergency department*[tiab] OR emergency room*[tiab] OR emergency unit*[tiab] OR ward*[tiab] OR emergency outpatient unit*[tiab] OR trauma center*[tiab] OR trauma centr*[tiab] | 1405045 |
| #1 | "Bariatrics"[Mesh] OR "Bariatric Surgery"[Mesh] OR "Anastomosis, Roux-en-Y"[Mesh] OR bariatric*[tiab] OR obesity surger*[tiab] OR metabolic surger*[tiab] OR stomach stapling*[tiab] OR gastric bypass*[tiab] OR gastroileal bypass*[tiab] OR gastrojejunostom*[tiab] OR roux-en-y[tiab] OR LRYGB[tiab] OR RYGB[tiab] | 37805 |

## **Embase.com History October 17, 2019**

| Search | Embase.com Query October 17, 2019 | Items found |
| --- | --- | --- |
| #4 | #3 NOT 'conference abstract'/it | 6483 |
| #3 | #1 AND #2 | 11882 |
| #2 | 'hospitalization'/exp OR 'emergency health service'/exp OR 'hospital readmission'/exp OR hospital*:ab,ti,kw OR rehosp*:ab,ti,kw OR readmission*:ab,ti,kw OR (emergency NEAR/3 service*):ab,ti,kw OR (emergency NEAR/3 department*):ab,ti,kw OR (emergency NEAR/3 room*):ab,ti,kw OR (emergency NEAR/3 unit*):ab,ti,kw OR ward*:ab,ti,kw OR (trauma NEAR/3 center*):ab,ti,kw OR (trauma NEAR/3 centr*):ab,ti,kw | 2152598 |
| #1 | 'bariatrics'/exp OR 'bariatric surgery'/exp OR 'Roux Y anastomosis'/exp OR 'gastric bypass surgery'/exp OR bariatric*:ab,ti,kw OR (obesity NEAR/3 surger*):ab,ti,kw OR (metabolic NEAR/3 surger*):ab,ti,kw OR (stomach NEAR/3 stapling*):ab,ti,kw OR ‘gastr* bypass*’:ab,ti,kw OR gastrojejunostom*:ab,ti,kw OR ‘roux-en-y’:ab,ti,kw OR LRYGB:ab,ti,kw OR RYGB:ab,ti,kw | 66853 |

## **PsycINFO (Ebsco) Search History October 17, 2019**

| Search | PsycINFO (Ebsco) Query - October 17, 2019 | Items found |
| --- | --- | --- |
| S3 | S1 AND S2 | 159 |
| S2 | DE ("Hospital Admission" OR "Hospitalization" OR "Emergency Services") OR TI (hospital* OR rehosp* OR readmission* OR emergency service* OR emergency department* OR emergency room* OR emergency unit* OR ward* OR trauma center* OR trauma centr*) OR AB (hospital* OR rehosp* OR readmission* OR emergency service* OR emergency department* OR emergency room* OR emergency unit* OR ward* OR trauma center* OR trauma centr*) OR KW (hospital* OR rehosp* OR readmission* OR emergency service* OR emergency department* OR emergency room* OR emergency unit* OR ward* OR trauma center* OR trauma centr*) | 178067 |
| S1 | DE "Bariatric Surgery" OR TI (bariatric* OR obesity surger* OR metabolic surger* OR stomach stapling* OR gastr* bypass* OR gastrojejunostom* OR “roux-en-y” OR LRYGB OR RYGB) OR AB (bariatric* OR obesity surger* OR metabolic surger* OR stomach stapling* OR gastr* bypass* OR gastrojejunostom* OR “roux-en-y” OR LRYGB OR RYGB) OR KW (bariatric* OR obesity surger* OR metabolic surger* OR stomach stapling* OR gastr* bypass* OR gastrojejunostom* OR “roux-en-y” OR LRYGB OR RYGB) | 1695 |

## **Search History Cochrane Library October 17, 2019**

| Search | Cochrane Library Query - October 17, 2019 | Items found |
| --- | --- | --- |
| #3 | #1 and #2 | 975 |
| #2 | hospital* or rehosp* or readmission* or (emergency near/3 service*) or (emergency near/3 department*) or (emergency near/3 room*) or (emergency near/3 unit*) or ward* or (trauma near/3 center*) or (trauma near/3 centr*) :ti,ab,kw (word variations have been used) | 171933 |
| #1 | (bariatric* or (obesity near/3 surger*) or (metabolic near/3 surger*) or (stomach near/3 stapling*) or (gastr* near/3 bypass*) or gastrojejunostom* or "roux-en-y" or LRYGB or RYGB):ti,ab,kw (word variations have been used) | 4902 |

## **Search History Scopus October 17, 2019**

| Search | Scopus Query - October 17, 2019 | Items found |
| --- | --- | --- |
| #3 | #1 AND #2 | 7536 |
| #2 | TITLE-ABS-KEY(hospital* OR rehosp* OR readmission* OR (emergency W/3 service*) OR (emergency W/3 department*) OR (emergency W/3 room*) OR (emergency W/3 unit*) OR ward* OR (trauma W/3 center*) OR (trauma W/3 centr*)) | 2301979 |
| #1 | TITLE-ABS-KEY(bariatric* OR (obesity W/3 surger*) OR (metabolic W/3 surger*) OR (stomach W/3 stapling*) OR (gastr* PRE/0 bypass*) OR gastrojejunostom* OR (roux PRE/0 en PRE/0 y) OR LRYGB OR RYGB) | 43667 |
